# Heterozygous *P32*/*C1QBP*/*HABP1* Polymorphism *rs56014026* Reduces Mitochondrial Oxidative Phosphorylation and Is Expressed in Low-grade Colorectal Carcinomas

**DOI:** 10.3389/fonc.2020.631592

**Published:** 2021-02-08

**Authors:** Annika Raschdorf, Annika Sünderhauf, Kerstin Skibbe, Berhane Ghebrehiwet, Ellinor I. Peerschke, Christian Sina, Stefanie Derer

**Affiliations:** ^1^Institute of Nutritional Medicine, University Hospital Schleswig-Holstein, Campus Lübeck, Lübeck, Germany; ^2^Department of Medicine, Stony Brook University, Stony Brook, NY, United States; ^3^Department of Laboratory Medicine, Memorial Sloan Kettering Cancer Center, New York, NY, United States; ^4^1^st^ Department of Medicine, Division of Nutritional Medicine, University Hospital Schleswig-Holstein, Campus Lübeck, Lübeck, Germany

**Keywords:** colorectal cancer, metabolism, mitochondria, OXPHOS, p32, single nucleotide polymorphism, C1QBP

## Abstract

Rapid proliferation of cancer cells is enabled by favoring aerobic glycolysis over mitochondrial oxidative phosphorylation (OXPHOS). P32 (*C1QBP*/gC1qR) is essential for mitochondrial protein translation and thus indispensable for OXPHOS activity. It is ubiquitously expressed and directed to the mitochondrial matrix in almost all cell types with an excessive up-regulation of p32 expression reported for tumor tissues. We recently demonstrated high levels of non-mitochondrial p32 to be associated with high-grade colorectal carcinoma. Mutations in human p32 are likely to disrupt proper mitochondrial function giving rise to various diseases including cancer. Hence, we aimed to investigate the impact of the most common single nucleotide polymorphism (SNP) *rs56014026* in the coding sequence of *p32* on tumor cell metabolism. *In silico* homology modeling of the resulting p.Thr130Met mutated p32 revealed that the single amino acid substitution potentially induces a strong conformational change in the protein, mainly affecting the mitochondrial targeting sequence (MTS). *In vitro* experiments confirmed an impaired mitochondrial import of mutated p32-T130M, resulting in reduced OXPHOS activity and a shift towards a low metabolic phenotype. Overexpression of p32-T130M maintained terminal differentiation of a goblet cell-like colorectal cancer cell line compared to p32-wt without affecting cell proliferation. Sanger sequencing of tumor samples from 128 CRC patients identified the heterozygous SNP *rs56014026* in two well-differentiated, low proliferating adenocarcinomas, supporting our *in vitro* data. Together, the SNP *rs56014026* reduces metabolic activity and proliferation while promoting differentiation in tumor cells.

## Introduction

One of the major differences between differentiated and proliferating tissues is their cell metabolism as a result of different metabolic requirements of the cells. While differentiated post-mitotic cells maintain their energy level primarily *via* mitochondrial oxidative phosphorylation (OXPHOS), most proliferating cells rely on aerobic glycolysis for energy production (1). Although much less energy efficient than OXPHOS (up to 36 mole ATP per mole glucose), aerobic glycolysis (approximately 4 mole ATP per mole glucose) can rapidly provide macromolecular precursors for anabolic pathways needed for cell division (2).

The metabolic switch from gaining energy *via* balanced OXPHOS towards aerobic glycolysis, or the so-called Warburg effect, is considered to be an important driver of proliferation and tumor formation (1, 3–5).

Initially, it was proposed that tumor cells manifest a mitochondria dysfunction (4), but in contrast to prior assumption it has been shown that functional mitochondria are essential for rapid cancer cell proliferation (6, 7). ATP production by OXPHOS is required for tumors to progress *in vivo* (8, 9) and some cancer cell lines have even revealed to mainly depend on OXPHOS for ATP supply under normoxia (10, 11). The question whether mutations affecting mitochondrial function promote or inhibit colorectal tumor growth is still controversial. A study by Ericson *et al*. reported the frequency of mitochondrial mutations to be decreased in colorectal cancer relative to normal tissues, suggesting that mutagenesis in mitochondrial DNA (mtDNA) is disadvantageous for tumor development and may even impede it (12). Conversely, a recent study indicated accumulation of mtDNA mutations in colon cancer to cause OXPHOS dysfunction and metabolic rewiring characterized by specific upregulation of the *de novo* serine synthesis pathway, conferring a distinct metabolic advantage for tumor growth (13).

The single nucleotide polymorphism (SNP) *rs56014026* is the most prominent SNP in the coding sequence (CDS) of the gene *P32* on chromosome 17 p13.2. Full-length p32 (282 aa) possesses an *N*-terminal mitochondrial targeting sequence (MTS) directing the protein to the mitochondria (14), although it has also been found in the cytosol (15), the nucleus (15) on the cell surface (16) or in the extracellular compartment (17). Apart from an enormous functional diversity in the fields of inflammation and infection, in recent years, p32 has emerged to play a pivotal role in the overall growth, survival and metastasis of tumor cells (14). Studies have shown that most tumor types exhibit increased expression levels of p32 (18), which often correlate with tumor stage and poor prognosis in cancer patients (19–23). For colorectal carcinomas we recently found that non-mitochondrial p32 is associated with increasing tumor grade (24). Until now, it is assumed that mitochondrial p32 is essential for mitochondrial protein synthesis, thereby critically maintaining OXPHOS, as complexes I, III, IV and V of the electron transport chain (ETC) contain subunits encoded by the mtDNA (14, 25, 26).

Short-hairpin RNA (shRNA)-induced knockdown of p32 in human breast cancer cells has been shown to reduce total oxygen consumption by about one third with concomitant increase in glycolysis, resulting in decreased cell proliferation and tumorigenicity *in vivo*. When p32 expression was restored to the original level in the knockdown cells, metabolic phenotype, proliferation rate and tumorigenicity could be rescued (25). By virtue of its regulatory impact on mitochondria activity, we hypothesized that mutations in p32 are likely to disrupt balanced cell metabolism giving rise to various diseases including cancer.

The SNP *rs56014026* indicates the exchange of cytosine (C) by thymine (T) at nucleotide position 389 of the human *p32* mRNA, resulting in substitution of threonine at amino acid position 130 into methionine (p.Thr130Met) in p32. Given its pivotal role for mitochondrial function, we aimed to decipher the impact of the SNP *rs56014026* on tumor cells’ metabolism and differentiation in the context of colorectal cancer.

## Materials and Methods

### Study Population

Complementary DNA (cDNA) samples derived from tumor tissues of CRC patients utilized in Sanger sequencing experiments were purchased from OriGene Technologies Inc. (Rockville, MD, USA). The gender- and age-matched cohort comprised 128 CRC patients (59 male, 69 female) with a median age [± SD] of 70 [± 13.51] years. Diagnosed tumors ranged from well differentiated G1 to undifferentiated G4 adenocarcinomas, being classified as stage I to IV. Twenty matched RNA samples from tumor and normal tissue of ten CRC patients analyzed by qPCR experiments depicted in heatmaps were purchased from OriGene Technologies Inc. The cohort was gender- and age-matched with a median age [± SD] of 74.50 [± 8.45] years and comprised ten G1 or G2 adenocarcinomas of the colon, all classified as stage IIA. Detailed patients’ characteristics are depicted in **Table 1**.

**Table 1:** Overview of study population.

		Male[n]	Female [n]	Median age [y]	TNM[n]	Tumor stage [n]	Tumor grade [n]	Tumor cells [%]
**Tumor samples from CRC patients for Sanger sequencing** [n=128]	tumor	59	69	male: 68 female: 72	pT1pN0pMX [3] pT1pN1pMX [3] pT1pN1pM1 [1] pT2pN0pMX [17] pT2pN1pMX [2] pT2pN2pMX [3] pT2pN1pM1 [1] pT3pN0pM0 [1] pT3pN0pMX [36] pT3pN0pM1 [4] pT3pN1pMX [15] pT3pN1pM1 [3] pT3pN2pMX [10] pT3pN2pM1 [3] pT4pN0pMX [9] pT4pN0pM1 [1] pT4pN1pMX [7] pT4pN2pMX [4] pT4pN2pM1 [2] pTXpNXpM1 [3]	I [19] II [1] IIA [37] IIB [9] III [7] IIIA [4] IIIB [17] IIIC [16] IV [18]	G1 [52] G2 [54] G3 [16] G4 [2] not reported [4]	72 (40–98)
**Paired samples from CRC patients for qPCR** [n=10]	normal tumor	5 5	5 5	74.5	n.a. pT3pN0pMX [10]	n.a. IIA [10]	n.a. G1 [3] G2 [7]	n.a. 73 (60–89)

CRC, colorectal carcinoma; n, number of patients; n.a., not applicable.

### Sanger Sequencing

To screen for the SNP *rs56014026* in *p32* transcripts, 128 colonic tumor samples collected from CRC patients (OriGene Technologies, Rockville, MD, USA; see **Table 1**) were analyzed by Sanger sequencing. Therefore, part of the *p32* cDNA was amplified by PCR using the oligonucleotides *p32*_forward: 5′-CTGCACACCGACGGAGACAA-3′ and *p32*_reverse: 5′-CATATAAGGCCCAGTCCAAG-3′. Sanger sequencing of amplicons was performed by Eurofins Genomics GmbH using the oligonucleotide *p32*_reverse.

For sequencing of *p32* transcripts in paired normal and tumor samples from ten CRC patients (OriGene Technologies; see **Table 1**), *p32* cDNA was amplified by PCR using the oligonucleotides *p32*_nt1_forward: 5′-ATGCTGCCTCTGCTGCG-3′ and *p32*_reverse. Subsequently, amplicons were Sanger sequenced by Eurofins Genomics GmbH using the oligonucleotides *hp32*_nt1_forward and *hp32*_reverse.

### Cell Culture

The human chronic myelogenous leukemia cell line HAP1-p32^−/−^ with a CRISPR/Cas9 induced knockout for *P32* (Horizon Discovery, Cambridge, UK) was cultivated in IMDM medium and the human colorectal carcinoma cell lines HT29 (American Type Culture Collection (ATCC), Manassas, VA, USA) and HT29-MTX-E12 (Sigma-Aldrich, St. Louis, MO, USA) were kept in DMEM medium. Both cell culture media were supplemented with 10% (v/v) FBS, 100 U/ml penicillin and 100 µg/ml streptomycin. Additionally, 1% non-essential amino acids (NEAA) was added to the medium for HT29-MTX-E12 cells. Cells were incubated at 37°C and 5% CO_2_ in a humidified incubator and confirmed to be negative for mycoplasma contamination every three months. For experiments, cells have been cultivated up to a maximum of 20 passages.

### Site-directed Mutagenesis of Human p32

The expression plasmid pCMV3-p32 for human wild type (wt) *p32* (Sino Biological, Beijing, China) was utilized for substitution of cytosine (C) at nucleotide position 389 by thymine (T) using the Quik Change II XL site-directed mutagenesis kit (Agilent, Santa Clara, CA, USA) and the mutagenic oligonucleotides *p32*_T130M_forward: 5′-GTTGCCGGGGAAAAAATCATGGTCACTTTCAACATTAACAACAGC-3′ and *p32*_T130M_reverse: 5′-GCTGTTGTTAATGTTGAAAGTGACCATGATTTTTTCCCCGGCAAC-3′, resulting in substitution of threonine (T) at amino acid position 130 by methionine (M) in p32. The mutated plasmid was Sanger sequenced by Eurofins Genomics GmbH (Ebersberg, Germany) using the oligonucleotide *p32*_exon2_forward: 5′-ATGTCTGGAGGTTGGGAG-3′.

### Transfection of Cell Lines

For stable transfection, HAP1-p32^−/−^ cells were seeded at a density of 0.8 × 10^6^ cells per well in a 6-well-plate in IMDM medium supplemented with 10% (v/v) FBS. Twenty-four hours after seeding, knockout cells were transfected with plasmids encoding the sequences for p32-wt or p32-T130M or with an empty vector by lipofection using the Lipofectamine™ 3000 kit (Thermo Fisher Scientific, Waltham, MA, USA), according to the manufacturer’s instructions. Twenty-four hours after transfection, cells were put under selection by adding 200 µg/ml Hygromycin B (Thermo Fisher Scientific). For transient transfection, HT29-MTX cells were reverse transfected with p32-wt or p32-T130M plasmids or with an empty vector at a density of 0.5 × 10^6^ cells per well. Cells were cultivated for 72 h in DMEM medium supplemented with 10% (v/v) FBS.

### Growing of Spheroids

Stable HAP1 transfectants were grown as spheroids using the hanging drop technique. Drops with a volume of 30 µl IMDM medium supplemented with 10% (v/v) FBS, 100 U/ml penicillin, 100 µg/ml streptomycin and 200 µg/ml Hygromycin B containing 10,000 cells per drop were placed on the bottom side of the lid of a 10 cm cell culture dish. To minimize evaporation, 10 ml of supplemented IMDM medium were placed in the bottom of the dish. Spheroids were grown for 8 days and medium was changed on day 4 and 6. Finally, spheroids were imaged with the Axio Scope.A1 microscope (2.5× magnification; Carl Zeiss, Oberkochen, Germany) and the area of each spheroid was determined using the ImageJ software (National Institutes of Health, Bethesda, MD, USA).

### RNA Extraction and Real-time Quantitative PCR

RNA was isolated from cell pellets utilizing the innuPREP RNA Mini Kit 2.0 (Analytik Jena AG, Jena, Germany) according to the manufacturer’s instructions and transcribed to cDNA using the RevertAid H Minus Reverse Transcriptase (Thermo Fisher Scientific) and Oligo(dT)_18_ primers. Real-time quantitative PCR (qPCR) was performed using Perfecta SYBR Green FastMix (Quanta BioSciences Inc., Gaithersburg, MD, USA) plus specific oligonucleotides in a 96-well plate format. The amplification program consisted of (i) preincubation at 95°C for 5 min and (ii) 40 cycles of denaturation at 95°C for 45 sec, annealing at 55°C for 30 sec and elongation at 72°C for 30 sec using the StepOnePlus Real-Time PCR System (Thermo Fisher Scientific). The following oligonucleotides were used for analyses (*ATOH1*: for: 5′-CCAGCTGCGCAATGTTATCC-3′, rev: 5′-TGCTGTTTTCCTCCTGCACT-3′; *HES1*: for: 5′-CTACCCCAGCCAGTGTCAAC-3′, rev: 5′-GGTCACCTCGTTCATGCAC-3′; *Ki67*: for: 5′-CCTGCTTGTTTGGAAGGG-3′, rev: 5′-GCTGGCTCCTGTTCACGTAT-3′; *KLF4*: for: 5′-CCATCTTTCTCCACGTTCG-3′, rev: 5′-ATCGGATAGGTGAAGCTGCA-3′; *LDHa*: for: 5′-GCACCCAGTTTCCACCATGA-3′, rev: 5′-GCACTCTTCTTCAAACGGGC-3′; *LGR5*: for: 5′-CACACACTGTCATTGCGAG-3′, rev: 5′-GCTTCTGTGGGTACGTGTCTT-3′; *p32*: for: 5′-CTGCACACCGACGGAGACAA-3′, rev: 5′-CATATAAGGCCCAGTCCAAG-3′; *Slc2a1*: for: 5′-TGGCATCAACGCTGTCTTCT-3′, rev: 5′-CTAGCGCGATGGTCATGAGT-3′; *SPDEF1*: for: 5′-GATTCACTACTGTGCCTCGAC-3′, rev: 5′-ATGTCTGGCTTCCGGATGAT-3′; *β-actin*: for: 5′-ACATCCGCAAAGACCTGTACG-3′, rev: 5′-TTGCTGATCCACATCTGCTGG-3′). After melting curve profiling, amplification curves were analyzed according to the 2^-dCt^ algorithm and expression levels were normalized to *β-actin*.

Expression of complement components in paired CRC samples was additionally analyzed by Taqman probes according to manufacturer’s instructions using the StepOnePlus Real-Time PCR System. The following cycling conditions were applied: (i) preincubation at 50°C for 2 min and 95°C for 10 min and (ii) 40 cycles of denaturation at 95°C for 15 sec and annealing and elongation at 60°C for 1 min. The following Taqman probes (Thermo Fisher Scientific) were used: *C1QA* (Hs00381122_m1), *C1QB* (Hs00608019_m1), *C1QC* (Hs00757779_m1), *C1R* (Hs00357637_m1), *C1S* (Hs01043794_m1), *C2* (Hs00918862_m1), *C3* (Hs00163811_m1), *C4A* (Hs00416393_g1), *C5* (Hs00156197_m1), *C6* (Hs01110040_m1), *C7* (Hs00940408_m1), *CFB* (Hs00156060_m1), *C3AR1* (Hs00377780_m1), *C5AR1* (Hs00383718_m1), *C5AR2* (Hs00218495_m1), *CR1* (Hs00559348_m1), *CR2* (Hs00153398_m1), *P32* (Hs00241825_m1), *CD93* (Hs00362607_m1), *CD46* (Hs00611257_m1), *CD55* (Hs00892618_m1), *CD59* (Hs00174141_m1), *C4BPB* (Hs00361221_m1), *ITGAM* (Hs00355885), *ITGAX* (Hs00174217) and *ACTB* (Hs99999903_m1). Expression levels of complement transcripts determined *via* the 2^-dCt^ algorithm were normalized to *β-actin*.

### Cell Fractionation

For cell factionation of stable HAP1 transfectants, cell pellets were resuspended in 250 µl of cold non-denaturing lysis buffer containing 1% phosphatase inhibitor cocktail II (Th. Geyer, Renningen, Germany) and cells were disrupted by drawing cell suspensions up and down through a 26 G needle causing shear force. Cell lysates were fractionated by different successive centrifugation steps.

### Western Blot and Antibodies

Cell pellets were lysed by resuspension in denaturing lysis buffer containing Tris and SDS supplemented with 2% phosphatase inhibitor cocktail II (Th. Geyer) and 3 (Sigma-Aldrich) and 1% protease inhibitor cocktail (Sigma-Aldrich). Samples were heated at 100°C for 5 min, cooled on ice, mixed and treated twice by ultrasonication for 20 s. Supernatants were collected after centrifugation at 12,000 *x g* at 4°C for 15 min.

Ten to 20 µg of whole-protein extracts or protein fractions were separated by denaturing SDS-PAGE utilizing a 4% to 15% gradient gel (Bio-Rad Laboratories, Hercules, CA, USA) under reducing conditions.

After separation, proteins were transferred to a PVDF membrane (GE Healthcare, Chicago, IL, USA) using a Trans-Blot^®^ semi-dry transfer cell (Bio-Rad Laboratories). After blocking with 5% w/v non-fat milk in TBS buffer with 0.1% Tween 20 (T-TBS), membranes were probed with primary antibodies diluted 1:1,000 in 5% w/v non-fat milk or 5% w/v BSA at 4°C over night. Primary antibodies specific for human p32 (clone EPR8871, ab131284 or clone 60.11, ab24733; both from Abcam, Cambridge, UK), β-actin (Cell Signaling Technology (CST), Danvers, MA, USA, #4967), Tom20 (CST, #42406), AMPKα (CST, #2532), phospho-AMPKα (CST, #2535) or KLF4 (R&D Systems, Minneapolis, MN, USA, #AF3640) were utilized. The next day, membranes were incubated with the corresponding secondary antibody conjugated to horseradish peroxidase (HRP). Proteins were visualized by chemiluminescence. To determine similar transfer and equal loading, membranes were reprobed with an antibody specific for β-actin.

### Determination of Kinase Phosphorylation Levels

Relative level of phosphorylation at 37 kinase phosphorylation sites and two related proteins were detected in stable HAP1-p32-wt and HAP1-p32-T130M transfectants using the Proteome Profiler™ Human Phospho-Kinase Array Kit (R&D Systems, Minneapolis, MN, USA). For each set of membranes (A and B) 600 µg of protein isolated from the respective cell pellets (see *Western blot and antibodies*) were used and the array was performed according to the manufacturer’s instructions. Spot intensity was analyzed densitometrically using ImageJ and was normalized to the reference spots.

### Immunofluorescence Staining

Immunofluorescence staining was performed according to standard protocols. Briefly, paraformaldehyde-fixed and de-paraffinized slides of cell pellets were incubated with primary antibodies specific for human p32 (clone 60.11; Abcam) or HSP60 (#sc-13115; Santa Cruz Biotechnology, Dallay, TX, USA), washed and incubated with respective fluorochrome-labelled IgG secondary antibodies (HSP60: Alexa-Fluor 488 nm; p32: Alexa-Fluor 594 nm; both from Thermo Fisher Scientific). Afterwards, slides were counterstained with DAPI (Sigma-Aldrich).

### Extracellular Oxygen Consumption Assay

To determine respiration rates of HAP1 or HT29-MTX transfectants, consumption of extracellular oxygen was measured. Therefore, cells were seeded in a 96-well microtiter plate at a density of 1 × 10^5^ cells per well and incubated at 37°C and 5% CO_2_ in a humidified incubator for 4 to 6 h. Real-time measurement of oxygen consumption was performed using the MitoXpress Xtra Oxygen Consumption Assay (Agilent) according to the manufacturer’s instructions.

### Determination of Lactate Production

Measurement of lactate produced by HAP1 or HT29-MTX transfectants was performed using the L-Lactic acid assay kit (Megazyme, Bray, Ireland), according to the manufacturer’s instructions. Supernatants from indicated cells (HAP1 transfectants: 5 × 10^3^ cells per well in a 96-well microtiter plate, incubation at 37°C and 5% CO_2_ for 96 h under normoxia or hypoxia; HT29-MTX transfectants: 0.5 × 10^6^ cells per well in a 6-well plate, incubation at 37°C and 5% CO_2_ for 72 h) were diluted 1:20 in 1× PBS. Amounts of lactate measured were normalized to the viable cell mass determined in the corresponding neutral red assay or to cell counts, respectively.

### Cell Viability Assay

The neutral red cell viability assay was performed to determine the viable cell mass in HAP1-p32-wt, HAP1-p32-T130M and HAP1-mock cultures. 5 × 10^3^ cells per well were seeded into a 96-well microtiter plate and incubated at 37°C and 5% CO_2_ for 96 h. To determine the effect of oxygen depletion, transfectants were cultivated under normoxia with 21% oxygen or under hypoxia in an incubator providing hypoxic conditions of 2% oxygen for 72 h.

After incubation, cells were stained using a neutral red dye (Sigma-Aldrich) diluted 1:100 in IMDM for 2 h, washed and destained with a solution consisting of 50% pure ethanol, 49% bidistilled water and 1% pure acetic acid to release the incorporated dye into the supernatant. To analyze the neutral red dye uptake, absorbance was measured at 540 nm against a background absorbance of 690 nm in a spectrophotometer.

### Statistical Analysis

Data are displayed graphically and were statistically analyzed using GraphPad Prism version 6.0. Statistical significance was determined by appropriate statistical tests, which are indicated in the corresponding figure legends. Results are displayed as mean ± SD of at least three independent experiments. P-values were calculated and null hypotheses were rejected when p ≤ 0.05.

## Results

### The SNP *rs56014026* Is the Most Common Coding Mutation of *p32*

**Figure 1:**
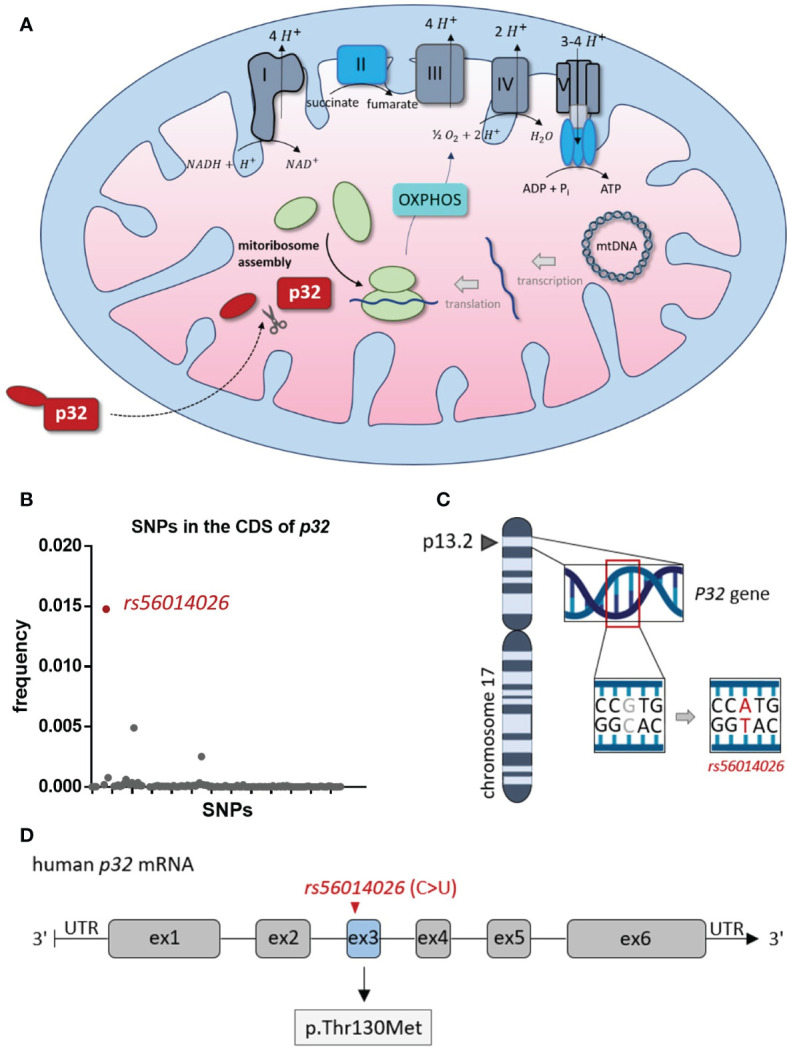
*Rs56014026* is the most common SNP in the CDS of P32. **(A)** Mitochondrial p32 functions in the assembly of the mitoribosome, which makes it essential for translation of the mitochondrially encoded subunits of the complexes I, III, IV and V of the electron transport chain (depicted in grey) [26, 27]. **(B)** Frequency of SNPs (n=124) located in the coding sequence (CDS) of human P32. Data are based on the SNP database (dbSNP) of the National Center for Biotechnology Information (NCBI). **(C)** The P32 gene is located at position 13.2 of the short (p) arm of human chromosome 17 (17p13.2). **(D)** Schematic model of human p32 mRNA (exons 1–6). The SNP *rs56014026*, leading to substitution of cytosine by uracil (C>U), is localized in exon 3 and results in the missense mutation p.Thr130Met (T130M) in p32.

Mitochondrial p32 plays an essential role in OXPHOS, as it functions in the assembly of the mitoribosome, thereby enabling translation of the mitochondrially encoded subunits of the complexes of the electron transport chain (26, 27) (**Figure 1A**). Among many other polymorphisms, *rs56014026* is the most common SNP in the CDS of *p32* with an estimated minor allele frequency (MAF) of 0.0148 (1.48%), as reported in the SNP database (dbSNP) of the National Center for Biotechnology Information (NCBI) (**Figure 1B**). The SNP *rs56014026* identifies the substitution of guanine by adenine at genome locus chr17:5434961 (GRCh 38.p12) in the *P32* gene (**Figure 1C**). Following transcription, the resulting cytosine to uracil exchange is located at nucleotide position 389 in exon 3 of the human *p32* mRNA, translating in the amino acid substitution p.Thr130Met (T130M) in p32 (**Figure 1D**).

### P.T130M Mutated p32 Decreases Mitochondrial OXPHOS Activity

**Figure 2:**
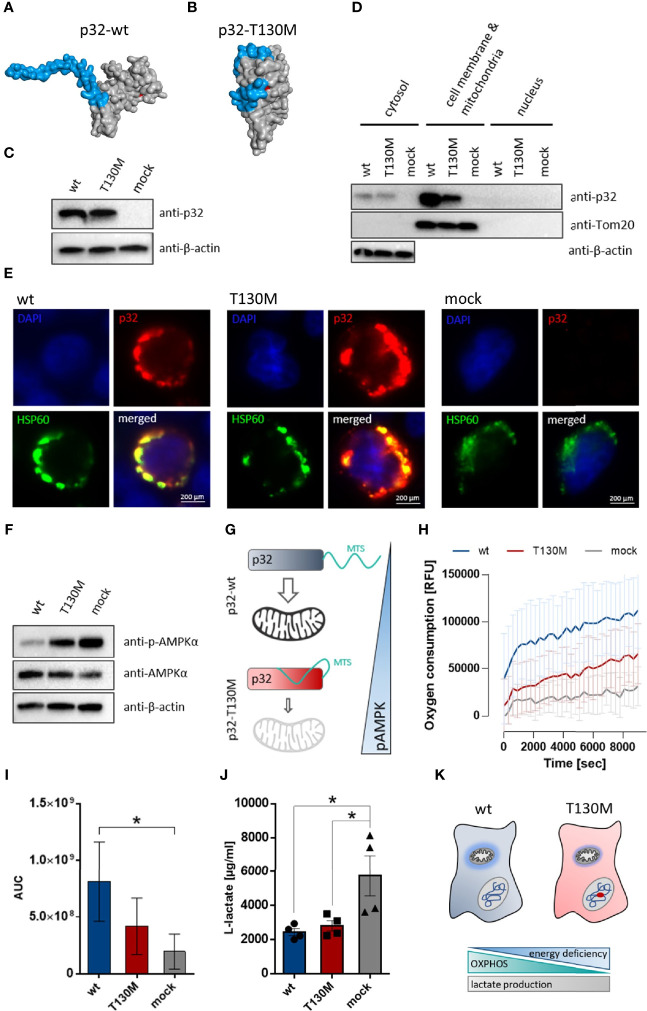
P32-T130M impairs mitochondrial OXPHOS activity in HAP1 cells. (A+B) Schematic structure model of the human **(A)** wild type and **(B)** T130M mutated p32 protein. Homology modeling of p32 was performed using Phyre2. The proteins are depicted as surface representations with the predicted mitochondrial targeting sequence (MTS) and amino acid 130 highlighted in blue and red, respectively. **(C)** Western blot experiment with whole protein extracts of stable HAP1-p32-wt, HAP1-p32-T130M and HAP1-mock transfectants was performed using the anti-p32 antibody clone 60.11 or an anti-β-actin antibody. **(D)** Cytosolic, mitochondrial/cell membrane and nuclear protein fractions of the stable HAP1 transfectants were analyzed by Western blotting using primary antibodies against p32 (clone 60.11), Tom20 or β-actin. **(E)** Representative fluorescence microscopy images of colocalization of p32 (antibody clone 60.11) with mitochondrial HSP60 protein in HAP1 transfectants. **(F)** Western blot analyses were performed with whole protein lysates of the stable HAP1 transfectants using primary antibodies against phospho-AMPKα, AMPKα or β-actin. **(G)** Impaired mitochondrial import of p32-T130M is accompanied by increased phospho-AMPK in HAP1 cells. MTS; mitochondrial targeting sequence **(H)** Time-dependent measurement of oxygen consumption of the stable HAP1 transfectants. **(I)** The AUC was calculated for each single experiment and each cell line. For statistical analysis of significance, Friedman test was performed followed by Dunn’s multiple comparison test. **(J)** L-lactate production was measured in cell culture supernatants from stable HAP1 transfectants after 96 h of incubation and normalized to the number of viable cells. Statistical significance was determined using a one-way ANOVA followed by Tukey’s multiple comparison test. **(K)** P.T130M mutated p32 decreases OXPHOS activity in HAP1 cells, while lactate production is unaffected. *p ≤ 0.05.

**Figure 3:**
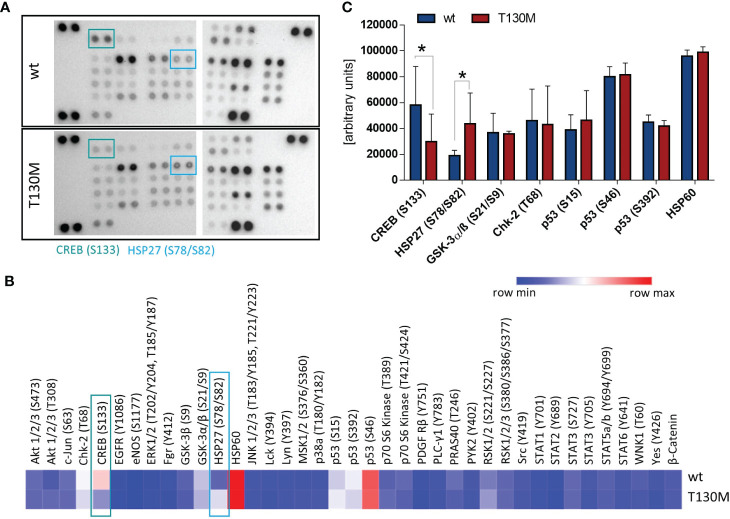
P32-T130M reduces CREB phosphorylation and induces HSP27 activation. **(A)** Relative levels of phosphorylation of 37 kinase phosphorylation sites and two related proteins were determined with the Human Phospho-Kinase Array using whole cell lysates of stable HAP1 transfectants. Representative image from two experiments is shown. **(B)** Intensity of spots was analyzed densitometrically using ImageJ with the mean of both experiments displayed in a heatmap. **(C)** Comparison of phosphorylation levels of the most highly activated proteins CREB, HSP27, GSK-3α/β, Chk-2, p53, and HSP60 between HAP1-p32-wt and HAP1-p32-T130M cells. S: Serine, T: Threonine, Y: Tyrosine. Statistical significance was calculated using a two-way ANOVA followed by Fisher’s LSD test. *p ≤ 0.05.

To model the impact of the polymorphism on protein structure, we performed homology modeling *in silico* using the Phyre2 server. Full-length human p32 protein (282 amino acids; 31.4 kDa) comprises an *N*-terminal targeting sequence for mitochondrial import (MTS), whose conformation was predicted to be strongly affected by the p.T130M mutation. While in wild type protein the MTS is accessible for mitochondrial import (**Figure 2A**), in mutated p32-T130M the conformation of the MTS is predicted to be altered, potentially preventing p32 binding to receptors for mitochondrial import (**Figure 2B**). Hence, we hypothesized that mitochondrial import of p32 may be reduced by the polymorphism *rs56014026*. To functionally analyze the effect of the polymorphism on mitochondrial import of p32 and possible consequences on cell metabolism *in vitro*, a plasmid encoding 389 C>U mutated *p32* was generated by a site-directed mutagenesis PCR using the plasmid encoding human wild type *p32*. Successful introduction of the SNP was verified by Sanger sequencing (**Supplementary Figure S1**). The near-haploid human chronic myelogenous leukemia (CML)-derived HAP1 cell line with a CRISPR/Cas9 induced knockout for *p32* (HAP1-p32^−/−^) was stably transfected with plasmids encoding wild type or mutated p32-T130M or with an empty vector. Successful transfection was visualized by Western blot experiments utilizing a primary antibody specific for human p32 (**Figure 2C**). Western blot experiments of fractionated cell lysates confirmed decreased mitochondrial import of mutated p32-T130M by displaying reduced amounts of p32 in the mitochondria/cell membrane fraction of HAP1-p32-T130M mutants compared to HAP1-p32-wt cells (**Figure 2D**). Of note, in cytosolic protein fractions no differences in p32 level were observed between p32-T130M and p32-wt transfectants. Additionally, co-localization of p32 and the mitochondrial heat shock protein 60 (HSP60) was assessed using immunofluorescence microscopy. While p32-wt was mainly localized to the mitochondria, mitochondrial localization of p32-T130M was decreased (**Figure 2E**). Notably, HAP1-mock transfectants depicted diminished mitochondrial mass as reflected by reduced and more diffuse HSP60 staining compared to the HAP1-p32^−/−^ cells transfected with *p32*. To investigate the consequence of diminished mitochondrial p32 localization on cell metabolism, we performed Western blot experiments to determine the phosphorylation state of 5′-AMP-activated protein kinase (AMPKα). AMPKα acts as a cellular energy sensor, as it is phosphorylated by sensing increases in the ratios of AMP/ATP and ADP/ATP and hence indicates energy deficiency in cells. In response to phosphorylation, it regulates energy balance by activating catabolic and downregulating anabolic pathways (28). Highest AMPKα activation was observed in mock transfected cells lacking p32 with decreasing activation level in p32-T130M mutants and lowest one in p32-wt cells (**Figure 2F**). Hence, decreased mitochondrial import of p.T130M mutated p32 was accompanied by an energy deficiency in HAP1 cells (**Figure 2G**). In line, oxygen consumption of p32-T130M mutants was decreased compared to p32-wt cells (**Figure 3H**). As expected, HAP1 cells deficient for p32 showed the lowest oxygen consumption with the area under the curve (AUC) displaying a significant 76% reduction compared to the p32-wt cells (**Figure 2I**). In the course of aerobic glycolysis, L-lactate is built from pyruvate by the lactate dehydrogenase (LDH) and is secreted into the extracellular compartment. HAP1-p32-T130M and HAP1-p32-wt transfectants displayed similar lactate production ± SEM of 2,800 ± 298.6 µg/ml and 2,400 ± 206.4 µg/ml, respectively, while p32 deficient cells exhibited significantly higher lactate concentrations of an average ± SEM of 5,800 ± 1,180 µg/ml (**Figure 2J**). Notably, neither the p32-T130M nor the mock transfectants compensated energy deficiency by upregulating expression of the glucose transporter 1 (*Slc2a1*) or the glycolytic enzyme *LDHa* (**Supplementary Figures S2A, B**). These experiments demonstrated that HAP1 cells expressing p32-wt are highly energetic performing aerobic glycolysis, while in HAP1-p32-T130M cells OXPHOS is impaired. As mitochondrial dysfunction is not compensated by anaerobic glycolysis, HAP1-p32-T130M cells are reduced in their energy status, producing less ATP compared to p32-wt expressing HAP1 cells. (**Figure 2K**).

### P32-T130M Decreases CREB Phosphorylation and Increases HSP27 Activation

To unravel the impact of *rs56014026* associated energy deficiency on cellular signal transduction, we studied activation of different critical signaling pathways in the HAP1 transfectants. Therefore, we determined the phosphorylation level of 37 kinase phosphorylation sites and two related proteins (**Figures 3A, B**). Comparison of the most highly activated proteins in HAP1 cells revealed that phosphorylation of the transcription factor cAMP response element-binding protein (CREB) at serine 133 (S133) was significantly reduced in HAP1-p32-T130M transfectants (**Figure 3C**). As CREB is overexpressed and constitutively phosphorylated in a number of human cancers, promoting survival and proliferation *via* different pathways (29), p32-T130M expression potentially triggers tumor-inhibiting signaling pathways as a result of decreased CREB activation. In contrast, phosphorylation of the heat shock protein 27 (HSP27) at serine 78/82 (S78/S82) was significantly increased in p32-T130M compared to p32-wt expressing HAP1 cells. Phosphorylation of HSP27 is induced in response to a variety of cellular stress stimuli and has been shown to prevent apoptosis (30). Hence, increased HSP27 activation in energy deficient HAP1-p32-T130M cells may potentially result in a cytoprotective effect.

### P32-T130M Increases Glycolytic Rate and Reduces Cell Proliferation Under Hypoxia

**Figure 4:**
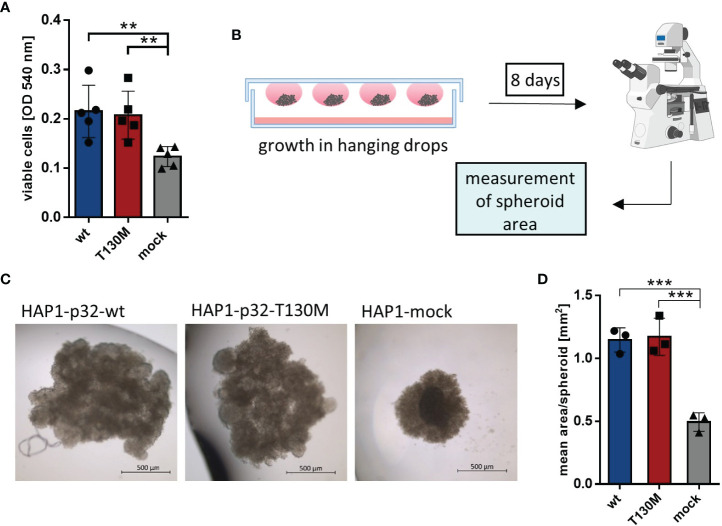
P32-T130M does not affect proliferation of HAP1 cells and growth of HAP1 cell-derived spheroids. **(A)** HAP1 transfectants were incubated for 72 h and cell viability was determined by a neutral red assay, measuring the optical density (OD) at 540 nm. **(B)** Graphical setup of HAP1 transfectants grown as spheroids in hanging drops. **(C)** Representative pictures of HAP1-*p32*-wt, HAP1-*p32*-T130M and HAP1-mock spheroids after 8 days of incubation (brightfield, 2.5× magnification). **(D)** Mean area of the HAP1 spheroids was determined using ImageJ. The three independent experiments comprise 10 to 12 spheroids each. For **(A)** and **(D)** statistical significance was determined using a one-way ANOVA followed by Tukey’s multiple comparison test. **p ≤ 0.01, ***p ≤ 0.001.

To analyze whether the detected mitochondrial dysfunction induced by expression of the polymorphism affects cell proliferation, HAP1 transfectants were incubated at normoxic conditions (21% oxygen) in a cell culture medium containing 25 mM glucose. Under normoxia, p32-T130M did not reduce cell proliferation compared to p32-wt cells, while the viable cell mass of HAP1 cells deficient for p32 was significantly decreased by 43% (**Figure 4A**). Additionally, HAP1 transfectants were grown in spheroids under normoxic conditions using the hanging drop technique, presenting an intermediate between monolayer cell culture and tumor growth *in vivo* (**Figure 4B**). HAP1-p32-wt and HAP1-p32-T130M transfectants formed significantly larger spheroids than HAP1-mock transfectants (**Figure 4C**). Calculation of the mean area of the spheroids showed that p32-wt and p32-T130M spheroids displayed the same size (mean ± SEM) of 1.15 ± 0.06 mm^2^ to 1.17 ± 0.09 mm^2^, while mock spheroids revealed a significantly smaller size of 0.49 ± 0.04 mm^2^ (**Figure 4D**). Similar to the results from monolayer cell culture, these experiments in 3D culture confirmed that p32-T130M mutants do not differ from p32-wt cells in cell proliferation under normoxic conditions.

**Figure 5:**
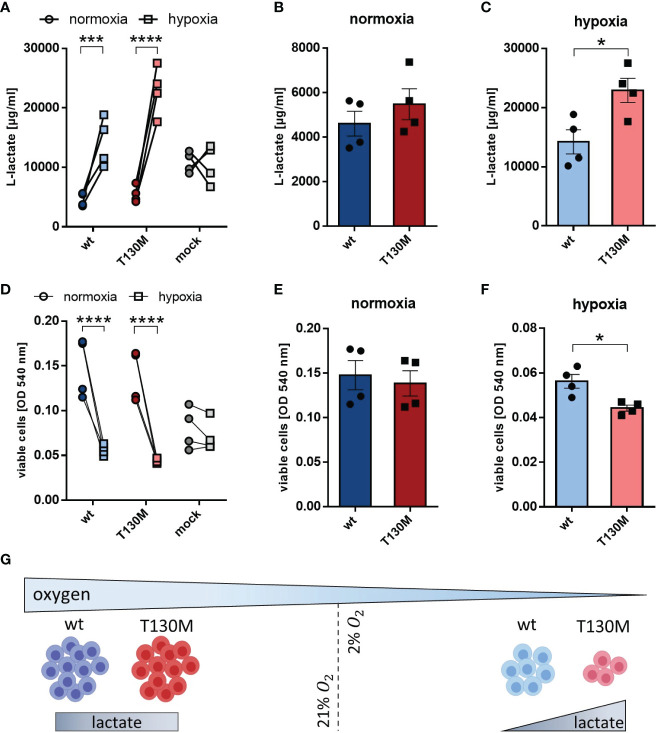
P32-T130M increases glycolysis and decreases proliferation of HAP1 cells under hypoxia. **(A–C)** HAP1 transfectants were cultivated under **(B)** normoxic or **(C)** hypoxic conditions for 72 h. l-lactate production was measured in cell culture supernatant and normalized to the number of viable cells. **(D–F)** Cell viability of HAP1 transfectants being cultivated for 72 h under **(E)** normoxia or **(F)** hypoxia was determined by a neutral red assay. **(G)** Under hypoxic conditions glycolysis is increased and cell proliferation is decreased for HAP1-p32-T130M compared to HAP1-p32-wt cells. For **(A**, **D)** and **(C**, **F)** statistical significance was determined using a two-way ANOVA followed by Sidak’s multiple comparison test or an unpaired t-test, respectively. *p ≤ 0.05, ***p ≤ 0.001, ****p ≤ 0.0001.

Since the majority of tumor cells are present in a hypoxic microenvironment, HAP1 cells were investigated under more physiological oxygen conditions. Under hypoxia (2% O_2_), lactate release of HAP1-p32-wt and HAP1-p32-T130M transfectants increased threefold from 4,602 ± 557 µg/ml to 14,208 ± 2,037 µg/ml or more than fourfold from 5,478 ± 694 µ/ml to 22,918 ± 2,048 µg/ml compared to normoxia (21% O_2_), respectively (**Figure 5A**). In the case of p32 deficient HAP1 cells no difference in lactate production was observed between normoxic or hypoxic conditions. Comparing p32-wt cells and p32-T130M mutants under normoxia, there was no significant difference in glycolytic rate (**Figure 5B**). Though, cultivation under hypoxia induced a significant increase in glycolysis in p32-T130M mutants (22,918 ± 2,048 µg/ml) in comparison to p32-wt cells (14,208 ± 2,037 µg/ml; **Figure 5C**).

Cell viability assays revealed that hypoxia significantly reduced proliferation of p32-wt and p32-T130M cells compared to normoxic conditions, while oxygen concentration had no impact on proliferation of HAP1 cells deficient for p32 (**Figure 5D**). Although cell proliferation did not differ between p32-T130M and p32-wt cells under normoxia (**Figure 5E**), it was reduced by 21% for p32-T130M mutants in comparison to p32-wt cells under hypoxia (**Figure 5F**). These experiments indicate that HAP1-p32-T130M cells shift more towards anaerobic glycolysis accompanied by reduced cell proliferation under physiological oxygen conditions compared to HAP1-p32-wt cells (**Figure 5G**). Hence, these data highlight the importance of efficient mitochondria function in cell proliferation.

### P32-T130M Decreases OXPHOS Activity and Promotes Differentiation in HT29-MTX Cells

**Figure 6:**
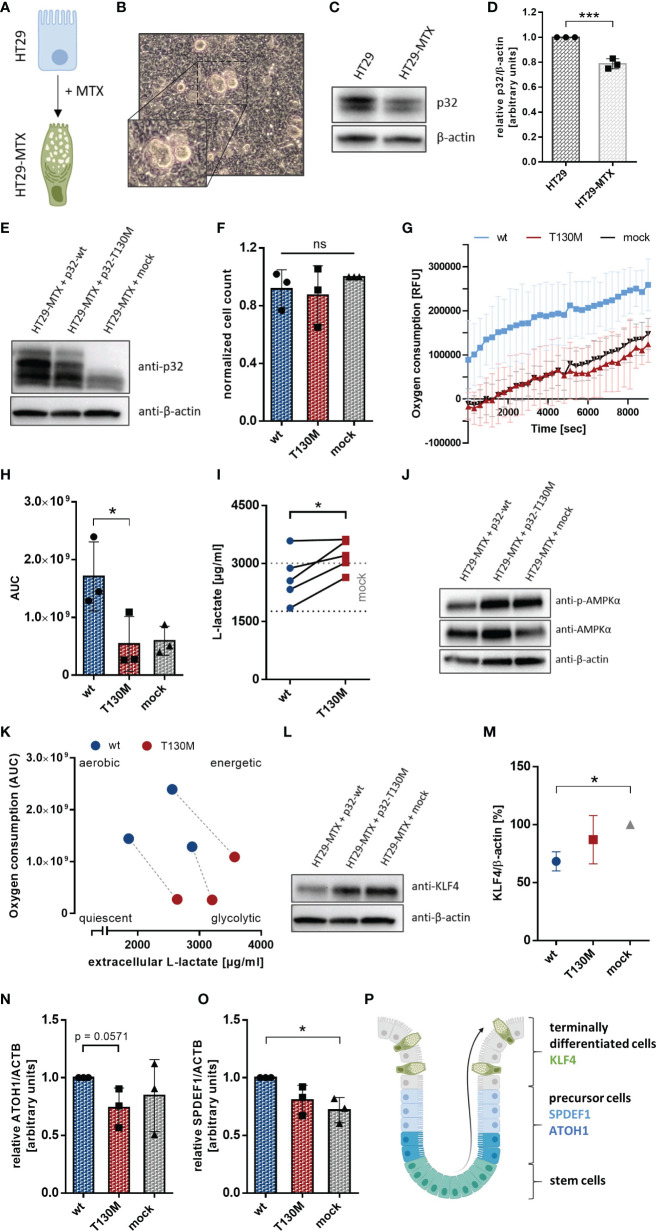
P32-T130M reduces OXPHOS activity and maintains efficient differentiation in HT29-MTX cells. **(A)** HT29-MTX cells derive from HT29 cells following differentiation induced by methotrexate (MTX). **(B)** Representative image of HT29-MTX cell growth characteristics displaying mucous vacuoles. **(C)** Protein level of p32 was compared between HT29 and HT29-MTX cells by Western blot experiments using primary antibodies against p32 (clone EPR8871) or β-actin. **(D)** Amount of p32 was quantified densitometrically using Western Blots of HT29 and HT29-MTX cell lysates. **(E)** Western blot experiment with whole protein extracts of transient HT29-MTX + p32-wt, HT29-MTX + p32-T130M and HT29-MTX + mock transfectants was performed using the anti-p32 antibody clone EPR8871. **(F)** Cell count of transient HT29-MTX transfectants was determined after 72 h of incubation and normalized to mock transfected HT29-MTX cells. **(G)** Time-dependent measurement of oxygen consumption of transient HT29-MTX transfectants. **(H)** The AUC was calculated for each single experiment and each cell line. **(I)** L-lactate production was measured in cell culture supernatants from transient HT29-MTX transfectants after 72 h of incubation and normalized to the cell count. Mean ± SD of HT29-MTX + mock transfectants is depicted by dotted gray lines. **(J)** Western blot experiment with whole protein extracts of transient HT29-MTX transfectants was performed using primary antibodies against phospho-AMPKα, AMPKα or β-actin. **(K)** Schematic representation of energy metabolism of HT29-MTX cells overexpressing p32-wt or p32-T130M using data displayed in **(H**, **I)**. Transfectants from the same experiment are connected by a dashed line. **(L)** Western blot analyses were performed with whole protein lysates of transient HT29-MTX transfectants using primary antibodies against KLF4 or β-actin. **(M)** For relative quantification bands were analyzed densitometrically using ImageJ and the amount of KLF4 was normalized to the amount of β-actin. **(N**, **O)** Expression of **(N)** ATOH1 and **(O)** SPDEF1 mRNA was quantified in transient HT29-MTX transfectants by qPCR. **(P)** Schematic model for goblet cell differentiation in the colonic crypt. Statistical analysis of significance for **(D**, **N**, **I)** was performed using an unpaired or paired t-test, respectively. For **(F**, **H**, **M**, **O)** statistical significance was determined using a one-way ANOVA followed by Tukey’s multiple comparison test. *p ≤ 0.05, ***p ≤ 0.001.

Since recent studies have shown that loss of mitochondrial activity drives colorectal tumor growth (13, 24), we further investigated the impact of the SNP *rs56014026* on the metabolism of the human colorectal carcinoma cell line HT29-MTX. HT29-MTX cells derive from the colon cancer cell line HT29 by differentiating into goblet cells under methotrexate (MTX) selection (31) (**Figure 6A**) and display numerous mucous vacuoles (**Figure 6B**). Differentiated HT29-MTX cells display reduced expression of endogenous p32 on protein level compared to parental HT29 cells, reflecting the reduced p32 expression described for low grade colorectal carcinomas (24) (**Figures 6C, D**). For the subsequent analyses, HT29-MTX cells were transiently transfected with plasmids encoding p32-wt, p32-T130M or with an empty vector and transfection efficiency was verified by Western blot experiments (**Figure 6E**). Counting of cells after 72 h of cultivation revealed no differences in cell proliferation between the transient transfectants (**Figure 6F**). Notably, overexpression of p32-wt induced a significant induction of OXPHOS activity, while overexpression of p32-T130M did not affect OXPHOS activity in comparison to mock transfected HT29-MTX cells (**Figure 6G**). Oxygen consumption of HT29-MTX + p32-T130M transfectants was significantly lower (−68%) in comparison to HT29-MTX + p32-wt transfectants (**Figure 6H**). Lactate production was increased in HT29-MTX cells overexpressing p.T130M mutated p32 (3,214 ± 182) compared to HT29-MTX cells overexpressing p32-wt (2,641 ± 290 µg/ml) or mock transfected cells (2,388 ± 278) (**Figure 6I**). As expected, p32-wt transfectants performing high OXPHOS had an increased energy level compared to p32-T130M and mock transfected cells, displayed by lower AMPKα phosphorylation (**Figure 6J**). Thus, p32-T130M overexpressing HT29-MTX cells turned out to be metabolically less active compared to p32-wt transfected HT29-MTX cells, partially compensating the lower OXPHOS rate by an increase in aerobic glycolysis (**Figure 6K**).

To study the impact of the polymorphism on cell differentiation, we performed Western blot experiments utilizing a primary antibody specific for human *Kruppel-like factor 4* (KLF4), a goblet cell-specific differentiation marker in the colon (32). P32-T130M overexpressing and mock transfected HT29-MTX cells showed increased expression of KLF4 in comparison to HT29-MTX cells overexpressing p32-wt (**Figure 6L**). Densitometric analysis revealed a reduction of KLF4 expression in p32-wt transfected cells of about 32% in comparison to mock transfected HT29-MTX cells (**Figure 6M**). In line with reduced KLF4 expression, mRNA expression of the precursor markers of the secretory lineage *Atonal Homolog 1* (*ATOH1*) and *SAM pointed domain-containing Ets transcription factor 1* (*SPDEF1*) (**Figure 6P**) was slightly increased in p32-wt compared to p32-T130M overexpressing HT29-MTX cells (**Figures 6N, O**). Since *SPDEF1* and *ATOH1* are markers for goblet cell progenitors, p32-T130M seems to maintain terminal goblet cell differentiation in contrast to overexpression of p32-wt. Hence, heterozygous expression of the polymorphism *rs56014026* results in reduced metabolic activity, characterized by balanced glycolysis and OXPHOS activities and thus in increased differentiation of HT29-MTX cells.

### Sequencing Study and Characterization of Human Colorectal Tumor Samples

**Figure 7:**
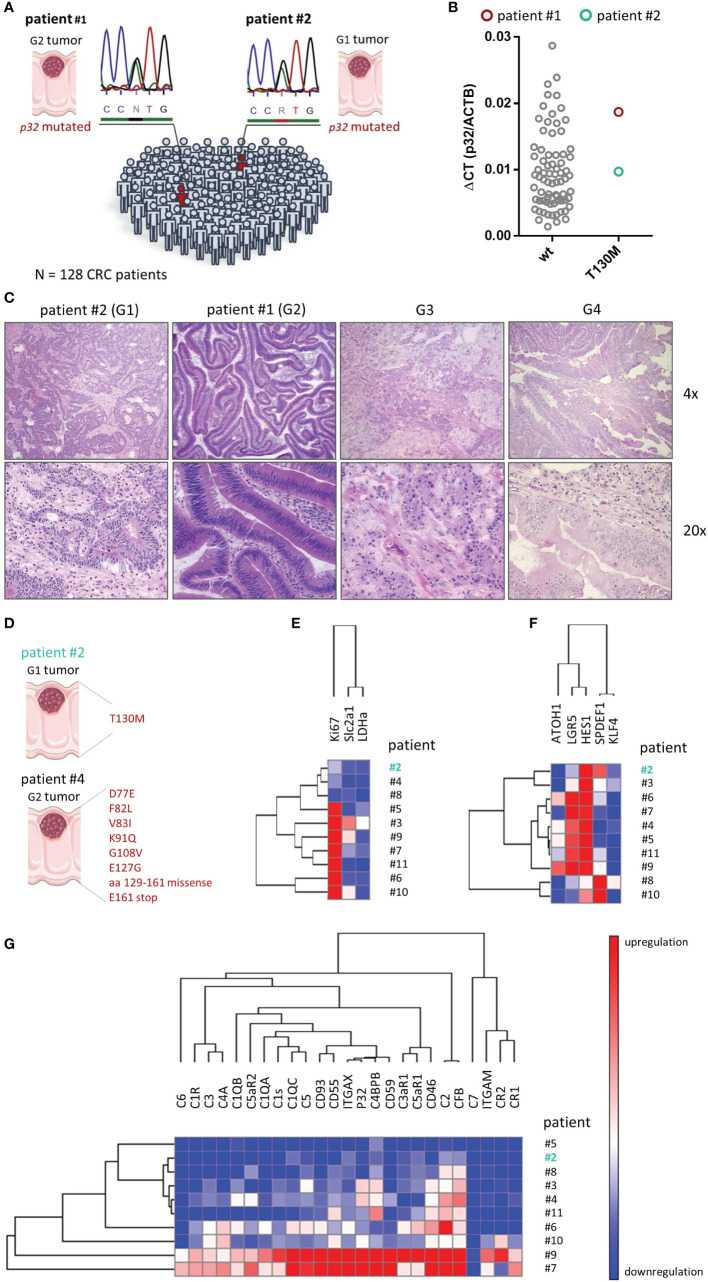
Screening for the SNP *rs56014026* in colon cancer patients. **(A)** The heterozygous SNP *rs56014026* was identified in two tumor samples of a total of 128 CRC patients by Sanger sequencing. Chromatograms depict sections of the reverse sequencing reactions. **(B)** *P32* mRNA expression in colorectal tumor samples encoding wt p32 (n=74) or heterozygous T130M mutated p32 (patient #1 and #2) was quantified by qPCR. **(C)** Pictures of hematoxylin and eosin (HE) staining of colonic tumor biopsies harboring the polymorphism collected from patient #2 (G1) and #1 (G2) as well as of a G3 and a G4 tumor (from OriGene Technologies). **(D)** Identified p32 mutations in cDNA samples from tumor tissues of patient #2 and #4. **(E)** Heatmap displaying mRNA expression levels of different metabolic proteins in ten colorectal tumor samples (patient #2 to #11) normalized to respective non-malignant colonic epithelium. **(F)** Heatmap showing mRNA expression levels of colonic differentiation markers in ten colorectal tumor samples (patient #2 to #11). **(G)** Quantification of mRNA expression of the components of the complement system in ten colorectal tumor samples (patient #2 to #11) normalized to respective non-malignant colonic epithelium. **(D**–**G)** Patient #2 (depicted in turquoise) carries the heterozygous SNP *rs56014026* in tumor, but not in normal tissue. Hierarchical clustering was performed by **(E)** city-block distance or **(F**, **G)** one minus Pearson correlation utilizing GENE-E (software.broadinstitute.org/GENE-E/).

The previous *in vitro* experiments suggested that the SNP *rs56014026* in *P32* shifts the metabolism of cancer cells into a more quiescent phenotype, accompanied by a decrease in the proliferation rate. To determine the rate of appearance of the SNP *rs56014026* in a pure CRC cohort, we Sanger sequenced *p32* in tumor samples of 128 CRC patients. The cohort comprised 59 male and 69 female CRC patients with a median age of 68 or 72 years, respectively, harboring poorly or well differentiated colorectal adenocarcinomas from grade 1 (G1) to grade 4 (G4) with staging between I and IV (**Table 1**). As expected from the MAF available in the dbSNP (1.48%; **Figure 1B**), we found the heterozygous SNP *rs56014026* in two out of 128 colorectal cancer patients (#1 and #2; 1.56%) being diagnosed with a G2 or G1 tumor, respectively (**Figure 7A**). Noteworthy, to determine and validate the frequency of this polymorphism in colorectal carcinoma patients more precisely, further studies utilizing larger CRC cohorts have to be performed. Both SNP *rs56014026* expressing tumors were classified as stage IIA and stage pT3pN0pMX according to the TNM staging system and were localized in the cecum. Comparing the expression of *p32* between tumors encoding wt or p.T130M mutated protein revealed no difference on mRNA level (**Figure 7B**). Reflecting the low grading of both tumors harboring the polymorphism, colonic tumor biopsies from patients #1 and #2 exhibit well defined epithelial structures in contrast to poorly differentiated G3 or G4 tumors (**Figure 7C**). To investigate the impact of the polymorphism on metabolism and differentiation of colorectal adenocarcinomas *ex vivo*, paired cDNA samples collected from normal and tumor tissue of ten CRC patients were analyzed by qPCR. While no mutation was detected in analyzed normal tissues, mutations in the coding sequence of *p32* were identified in paired tumor samples of CRC patient #2 and #4 (**Figure 7D**). While the tumor tissue of patient #2 expressed the heterozygous SNP *rs56014026*, the carcinoma of patient #4 exhibited multiple mutations (D77E, F82L, V83I, K91Q, G108V, E127G, aa 129 to 161 missense, E161 stop) in *p32*. One may hypothesize that the expression of non-functional p32 in the tumor of patient #4 will result in mitochondrial dysfunction and thus in a low energetic phenotype, similar to *P32* deficient cells (**Figures 2F–J**). First, we quantified expression of three key metabolic markers, with the expression in the tumor given as fold change compared to the respective normal tissue. As depicted by low *Ki-67* expression, the tumor harboring the polymorphism *rs56014026* (#2) was less proliferative compared to most of the other tumors (**Figure 7E**) and clustered with the tumor expressing non-functional p32 (#4) as well as with tumor #8. Reduced proliferative capacity of tumors #2, #4 and #8 might be explained by relatively low carbohydrate metabolism in these tumors, depicted by modest expression levels of lactate dehydrogenase A (*LDHa*) and solute carrier family 2, facilitated glucose transporter member 1 (*Slc2a1*), encoding the glucose transporter 1 (GLUT1). Further, we quantified the absolute expression levels of several colonic differentiation markers to determine the cellular origin of the tumors, as gene expression patterns were reported to be conserved during colorectal carcinogenesis (33). Most of the analyzed tumors displayed high expression of the stem cell marker *leucine-rich repeat-containing G-protein coupled receptor 5* (*LGR5*) as well as of *hairy and enhancer of split-1* (*HES1*), which is a marker of the absorptive epithelial cell lineage (**Figure 7F**). Reflecting the mucinous phenotype described for the tumors of patient #8 and #10, these tumors revealed high expression of the secretory progenitor marker *SPDEF1*, accompanied by low *LGR5* expression. Notably, tumor #2 exhibited only low *LGR5* intestinal stem cell marker expression, but consisted of substantial amounts of enterocytes and secretory progenitor cells as depicted by high *HES1* and *SPDEF1* as well as moderate *KLF4* expression, respectively. Hence, according to published data (33) one may conclude that composition of tumor #2 mostly resembled the cellular distribution found in normal colonic epithelium, suggesting that the tumor originated from the differentiated compartment in the upper part of the crypt rather than from the colonic stem cell compartment at the bottom of the crypt. Furthermore, p32 has also been characterized as a receptor for the globular heads of the complement component 1q (C1q) (34) and recent studies have reported that the presence of different factors of the complement system in the tumor microenvironment promote tumorigenesis (35–37). Products of the complement cascade have turned out to be major determinants of myeloid-derived suppressor cell recruitment into the tumor microenvironment, which promote tumor growth and create an immunosuppressive environment (38, 39). Hence, we additionally determined expression levels of complement components in these CRC samples. Therefore, mRNA expression of 25 complement components, receptors or inhibitors was studied utilizing target specific TaqMan arrays in qPCR experiments. As depicted in **Figure 7G**, expression of most complement system members was increased at least in some tumors, while mRNA of *C7* was reduced in each of the ten investigated adenocarcinomas compared to the respective normal tissue (**Figure 7G**). Complement components that were most frequently upregulated in tumor tissues were *CD46*, *C2* and *complement factor B* (*CFB*). Quantification revealed that expression of all analyzed compounds of the complement system was downregulated in the tumor tissue of patient #2 compared to most of the other tumors, suggesting that this tumor lacks high complement expression and associated pro-tumorigenic effects. In line with low *Ki-67* expression, the tumors of patient #2, #4 and #8 displayed low to moderate mRNA levels of the growth-promoting complement proteins C1q (40), p32 (41), CFB (42) as well as C3, C5, C3aR1 and C5aR1/2 (43, 44). Hence, analysis of the CRC cohort suggested that the SNP *rs56014026* is associated with differentiated G1 or G2 adenocarcinomas exhibiting low metabolic activity and complement expression, which potentially may explain the observed moderate cell proliferation in these colorectal tumors.

## Discussion

Tumor cell metabolism is characterized by a switch from balanced OXPHOS to aerobic glycolysis, allowing for rapid cell proliferation (1, 3–5). Mitochondrial p32 is pivotal for OXPHOS maintenance, as it essentially supports translation of the mitochondria encoded proteins of the complexes I, III, IV and V of the respiratory chain (14, 25, 26). In recent years, a crucial role for p32 in cancer has emerged, since expression is enhanced in most human cancer types, affecting growth, survival and metastasis (14). In this study the most common SNP in the CDS of *P32* (*rs56014026*) was analyzed to unravel potential effects on cell metabolism and thus on proliferation and tumor growth.

Our *in vitro* study revealed that mitochondrial import of p.T130M mutated p32 is impaired, potentially caused by a conformational change affecting the MTS, which leads to decreased OXPHOS activity. Respective cells showed an energy deficiency, resulting in a compensatory increase in glycolysis and reduced proliferation under hypoxia. By Sanger sequencing we found the polymorphism in two of 128 colorectal tumors, being characterized by low CRC grading and low expression of the cell proliferation marker *Ki-67*, which has to be further verified in larger patient cohorts.

While for many tumor types expression of p32 has been reported to correlate with tumor grade, stage and poor prognosis in patients (21, 24), we here propose the SNP *rs56014026* in *P32* to be associated with reduced proliferation and low grading in colorectal carcinomas through insufficient mitochondrial respiration. Although OXPHOS activity is strongly reduced by impaired mitochondrial import of mutated p32, aerobic glycolysis is not increased under optimal cell culture conditions, as cells probably do not have the need to optimize their energy metabolism. However, the polymorphism induces an increase in glycolysis under hypoxia, which is much closer to the tumor microenvironment *in vivo*, given that most solid tumors rapidly outgrow their blood supply (45). Thus, the heterozygous SNP *rs56014026* results in a shift from a highly energetic phenotype characterized by high OXPHOS and glycolysis activity to a more quiescent metabolic phenotype with only basal mitochondrial respiration. In line with OXPHOS being the major source of cellular energy, our data indicate that cancer cells carrying the heterozygous SNP *rs56014026* display an energy deficit, resulting in decreased proliferation under hypoxia. Additionally, we observed that mutated p32 is associated with enhanced differentiation in colorectal carcinoma cells *in vitro* and *ex vivo*, which supports the hypothesis, that the SNP is associated with low grading of colorectal tumors.

Despite metabolism being shifted towards aerobic glycolysis, mitochondrial OXPHOS is still essential in highly glycolytic cancer cells. Using [^13^C] glucose labeling, Scott *et al*. verified that metabolism of melanoma cells was not strictly glycolytic, even under hypoxia, as the tricarboxylic acid cycle was still functional Scott et al. (9). Moreover, as the expression of p32 is strongest in hypoxic regions within tumors, it is likely that p32 balances between OXPHOS and glycolysis to attenuate the otherwise detrimental switch to aerobic glycolysis (25).

As protein synthesis of p32 is not affected by the SNP *rs56014026* and mitochondrial import of p.T130M mutated p32 is impaired without concomitant increase in cytosolic protein, the question where the remaining extramitochondrial p32 is located instead awaits further investigation. Considering recent studies reporting different cancer cell lines to shed p32 into the extracellular compartment (46, 47), one could hypothesize that p.T130M mutated p32 may be increasingly secreted into the extracellular milieu.

Recently, our group suggested a mechanism for inflammation-driven carcinogenesis induced by caspase-1 cleavage of human p32 in response to NLRP3 inflammasome activation (24). In consequence of caspase-1–mediated cleavage of the *N*-terminal mitochondrial leader of p32, cell metabolism is shifted from balanced OXPHOS to excessive glycolysis activity boosting cell proliferation. Here, we show that cells encoding the SNP *rs56014026* compensate the reduction in OXPHOS only by a slight increase in glycolysis under oxygen depletion, giving rise to a rather quiescent metabolic phenotype with diminished proliferative capacity.

In many human cancer types overexpression and persistent activity of CREB promote survival and proliferation *via* upregulation of downstream genes, which leads to CREB being discussed as a target in cancer therapy (48). Interestingly, we could show that phosphorylation of CREB (S133) is significantly reduced in cancer cells carrying the SNP *rs56014026 in vitro*, further supporting an anti-tumorigenic potential of this polymorphism.

In conclusion, our data indicate that the heterozygous SNP *rs56014026* is disadvantageous for tumor growth while ensuring differentiation of tumor cells. The question whether changes in cell metabolism and signaling pathways induced by the polymorphism may in turn affect sensitivity towards certain chemotherapeutic drugs awaits further investigation. Although detailed impact of the SNP on tumor growth has not been identified *in vivo* yet, the present study highlights the significance of mutations in *P32* in the context of cancer metabolism. The functional analysis of this polymorphism opens up a broad field of research on many other SNPs in *P32* that may lead to similarly striking effects on tumor metabolism. This raises perspectives for new cancer treatment strategies targeting p32, potentially resulting in impaired mitochondrial energy production and cancer cell proliferation.

## Data Availability Statement

The original contributions presented in the study are included in the article/**Supplementary Material**, further inquiries can be directed to the corresponding author.

## Ethics Statement

Ethical review and approval was not required for the study on human participants in accordance with the local legislation and institutional requirements. Written informed consent for participation was not required for this study in accordance with the national legislation and the institutional requirements.

## Author Contributions

SD conceived the concept of the present study and supervised it. BG and EP provided the primary antibody 60.11 specific for human p32. AR and KS performed the experiments. AR and SD analyzed and interpreted the acquired data and drafted the article. AS, BG, CS, EP, and SD critically revised the article. All authors contributed to the article and approved the submitted version.

## Funding

This work was supported by the German Research Foundation (research grant DE 1874/1-2 to SD) and in part by the NIH/NCI Cancer Center Support Grant P30 CA008748. CS is Fresenius Kabi endowed professor of nutritional medicine.

## Conflict of Interest

The authors declare that the research was conducted in the absence of any commercial or financial relationships that could be construed as a potential conflict of interest with the exception of BG and EP who receive royalties from the sale of the monoclonal anti-p32 antibody 60.11.
