# Supplementary material for: Heterozygous P32/C1QBP/HABP1 Polymorphism rs56014026 Reduces Mitochondrial Oxidative Phosphorylation and Is Expressed in Low-grade Colorectal Carcinomas
Source: Front Oncol. 2021 Feb 8;10:631592. doi: 10.3389/fonc.2020.631592 (PMC7897657; doi:10.3389/fonc.2020.631592)
Supplement: Supplementary file 1 [file DataSheet_1.docx]

**Supplementary Figures**


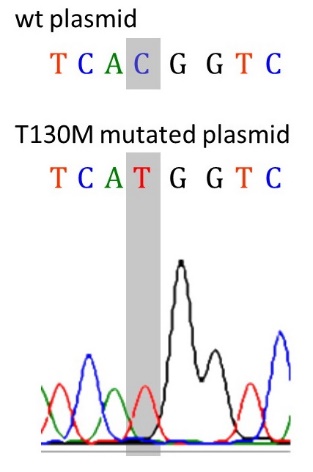


**Supplementary Figure S1.** Chromatogram of Sanger sequencing of the mutated plasmid exhibits thymine (T) at nucleotide position 389 of *p32* cDNA instead of cytosine (C) (highlighted in grey).


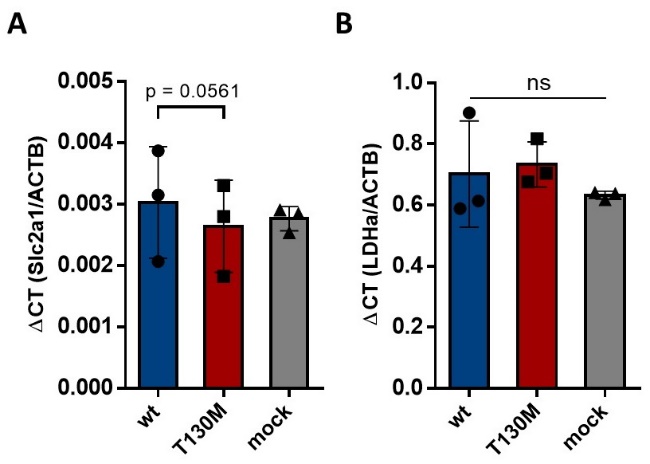


**Supplementary Figure S2.** Expression levels of (A) *Slc2a1* and (B) *LDHa* mRNA were quantified in stable HAP1-p32-wt, HAP1-p32-T130M and HAP1-mock transfectants by qPCR. Statistical significance was determined using (A) a paired t-test or (B) one-way ANOVA followed by Tukey’s multiple comparison test.
